# Smooth muscle cell specific NEMO deficiency inhibits atherosclerosis in ApoE^−/−^ mice

**DOI:** 10.1038/s41598-022-16737-8

**Published:** 2022-07-22

**Authors:** Takashi Imai, Trieu-My Van, Manolis Pasparakis, Apostolos Polykratis

**Affiliations:** 1 Institute for Genetics, University of Cologne, 50931 Cologne, Germany; 2 Centre for Molecular Medicine Cologne (CMMC), University of Cologne, 50931 Cologne, Germany; 3 Cologne Excellence Cluster on Cellular Stress Responses in Aging-Associated Diseases (CECAD), University of Cologne, 50931 Cologne, Germany; 4 CECAD Research Center, Institute for Genetics, University of Cologne, Joseph-Stelzmann-Str. 26, 50931 Cologne, Germany

**Keywords:** Cardiovascular biology, Inflammation

## Abstract

The development of atherosclerotic plaques is the result of a chronic inflammatory response coordinated by stromal and immune cellular components of the vascular wall. While endothelial cells and leukocytes are well-recognised mediators of inflammation in atherosclerosis, the role of smooth muscle cells (SMCs) remains incompletely understood. Here we aimed to address the role of canonical NF-κB signalling in SMCs in the development of atherosclerosis. We investigated the role of NF-κB signalling in SMCs in atherosclerosis by employing SMC-specific ablation of NEMO, an IKK complex subunit that is essential for canonical NF-κB activation, in *ApoE*^−*/*−^ mice. We show that SMC-specific ablation of NEMO (NEMO^SMCiKO^) inhibited high fat diet induced atherosclerosis in *ApoE*^−*/*−^ mice. NEMO^SMCiKO^/*ApoE*^−*/*−^ mice developed less and smaller atherosclerotic plaques, which contained fewer macrophages, decreased numbers of apoptotic cells and smaller necrotic areas and showed reduced inflammation compared to the plaques of *ApoE*^−*/*−^ mice. In addition, the plaques of NEMO^SMCiKO^/*ApoE*^−*/*−^ mice showed higher expression of α-SMA and lower expression of the transcriptional factor KLF4 compared to those of *ApoE*^−*/*−^ mice. Consistently, in vitro*,* NEMO-deficient SMCs exhibited reduced proliferation and migration, as well as decreased KLF4 expression and lower production of IL-6 and MCP-1 upon inflammatory stimulus (TNF or LPS) compared to NEMO-expressing SMCs. In conclusion, NEMO-dependent activation of NF-κB signalling in SMCs critically contributes to the pathogenesis of atherosclerosis by regulating SMC proliferation, migration and phenotype switching in response to inflammatory stimuli.

## Introduction

Atherosclerosis is a disease of the large arteries and the underlying cause of cardiovascular events such as heart attack and stroke. Inflammation is now recognised as a critical pathogenic factor in atherosclerosis^1–4^. Several studies highlighted the role of different cell types, including macrophages^5–7^, endothelial cells^6,8,9^, lymphocytes^10,11^, and smooth muscle cells (SMCs)^12,13^ in the initiation and progression of atherosclerotic lesions. Although the role of inflammation in the pathogenesis of atherosclerosis is well appreciated, recent studies revealed an increased complexity with inflammatory pathways exhibiting both proatherogenic and atheroprotective functions in different cell types^5,6,8^. Therefore, elucidating the relative contribution of different cell types in the inflammatory response controlling the initiation and progression of atherosclerosis will be crucial for understanding the mechanisms regulating the pathogenesis of atherosclerotic plaques and the development of more efficient therapeutic strategies.

The NF-κB signalling cascade regulates immune and inflammatory responses and is implicated in the development of atherosclerosis. NF-κB is the collective name for a family of transcription factors with five members: c-Rel, RelB, p65 (RelA), p105/p50, and p100/p52^14^. At steady state conditions NF-κB dimers are kept inactive in the cytoplasm by association with inhibitory proteins of the IκB family. Upon cell stimulation the IκB kinase (IKK) complex phosphorylates IκB proteins on specific serine residues targeting them for ubiquitination and proteasomal degradation. The released NF-κB dimers accumulate in the nucleus, where they activate the expression of many genes regulating inflammation. The IKK complex consists of two kinases, IKK1 (also known as IKKα) and IKK2 (also known as IKKβ) and a regulatory subunit named NF-κB essential modulator (NEMO, also known as IKKγ). NEMO is essential for IKK-mediated IκBα phosphorylation and activation of the canonical NF-κB signalling pathway, which primarily depends on IKK2 catalytic activity although the two kinases exhibit some degree of functional redundancy^15^.

Using mouse models allowing the cell-specific inhibition of NF-κB activation, we investigated previously the role of NF-κB in endothelial cells and macrophages in the development of atherosclerosis. We found that NF-κB inhibition in macrophages, achieved by myeloid cell-specific IKK2 ablation, resulted in increased atherosclerosis severity due to reduced levels of IL-10 expression and increased susceptibility of IKK2-deficient macrophages to cell death^5^. On the contrary, NF-κB inhibition specifically in endothelial cells achieved by either NEMO deficiency or transgenic expression of an IκBα super-repressor prevented the development of atherosclerotic plaques^8^. Interestingly, inhibition of TRAF6-dependent TLR signalling had similar effects, revealing that TLR signalling is proatherogenic in endothelial cells but atheroprotective in macrophages^6^. Together, these studies revealed an unexpected cell specificity of the role of TLR-mediated NF-κB signalling in atherosclerosis and underscored the necessity to dissect the role of inflammatory pathways in different cell types of the vascular wall in order to better understand the cellular and molecular mechanisms governing the pathogenesis of atherosclerosis.

In addition to endothelial and myeloid cells, SMCs are also implicated in the development of atherosclerotic plaques^16^. SMCs are the major producers of extracellular matrix within the vessel wall but also contribute to lipid uptake and the production of inflammatory mediators that attract immune cells to the developing plaques^16^. SMCs exhibit functional plasticity and have been reported to undergo phenotype switching adopting alternative phenotypes resembling macrophages, mesenchymal or osteochondrogenic cells, thus contributing positively or negatively on atherosclerotic plaque development^17–19^. However, the role of inflammatory signalling specifically in SMCs during the pathogenesis of atherosclerosis remains incompletely understood. Here we investigated the role of NF-κB signalling in SMCs in atherosclerosis by generating and analysing *ApoE*^−*/*−^ mice lacking NEMO specifically in SMCs. Our results revealed that inhibition of NEMO-dependent canonical NF-κB signalling in SMCs substantially inhibited the development of atherosclerotic plaques, demonstrating a critical pathogenic role of NF-κB signalling in SMCs in atherosclerosis.

## Results

### SMC-specific NEMO deficiency protects *ApoE*^−***/***−^ mice from atherosclerosis

**Figure 1:**
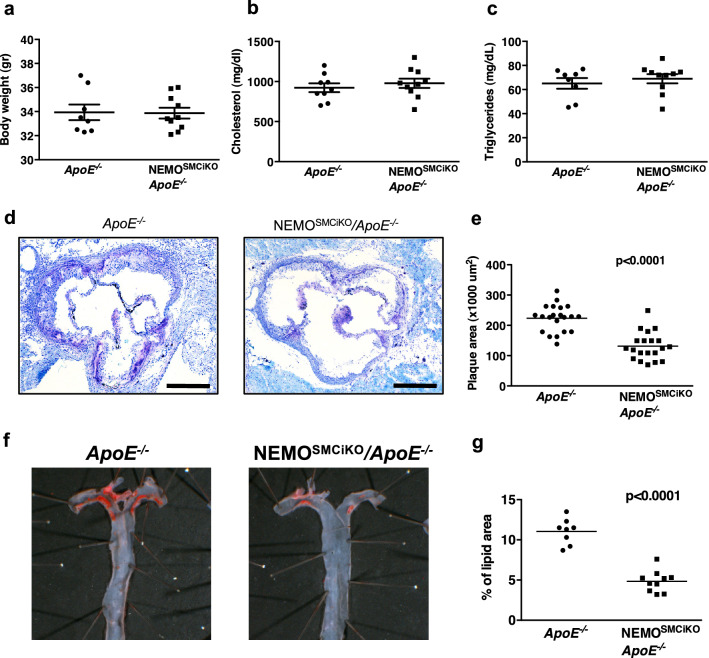
Inducible deletion of NEMO in smooth muscle cells inhibits atherosclerosis in *ApoE*^−*/*−^ mice. (**a**) Body weight (**b**) serum cholesterol levels and (**c**) serum triglyceride levels in *ApoE*^−*/*−^ (n = 8) and NEMO^SMCiKO^/*ApoE*^−*/*−^ (n = 10) mice after 10 weeks on HFD. (**d**) Representative aortal cross-sections from *ApoE*^−*/*−^ and NEMO^SMCiKO^/*ApoE*^−*/*−^ mice after 10 weeks on HFD*.* Scale bars = 0.5 mm. (**e**) Graph showing quantification of atherosclerotic lesion size at the aortic sinus of *ApoE*^−*/*−^ and NEMO^SMCiKO^/*ApoE*^−*/*−^ mice (Mann–Whitney test). (**f**) Representative *en face* staining in aortic arches from *ApoE*^−*/*−^ and NEMO^SMCiKO^/*ApoE*^−*/*−^ mice. (**g**) Graph showing quantification of the area of the aortas from *ApoE*^−*/*−^ and NEMO^SMCiKO^/*ApoE*^−*/*−^ mice that is covered with lipids (Mann–Whitney test).

To study in vivo the role of canonical NF-κB signalling in SMCs we generated mice with inducible SMC-restricted NEMO deficiency (NEMO^SMCiKO^) by crossing mice with loxP-flanked *Nemo* alleles^20^ to SMMH-CreER^T2^ mice^21^. Since the *Nemo* gene is located on the X chromosome and the SMMH-CreER^T2^ on the Y chromosome, only male mice from this line were suitable for our studies. In order to assess the role of SMC-specific NF-κB signalling in atherosclerosis, NEMO^SMCiKO^ mice were backcrossed into the ApoE-deficient genetic background. To induce ablation of NEMO in SMCs groups of mice were fed with a tamoxifen-containing chow diet for 6 weeks as previously described^6,8^. Tamoxifen-induced Cre activity resulted in NEMO ablation in SMCs in mice carrying both the SMMH-CreER^T2^ transgene and the loxP-flanked *Nemo* allele, while littermates carrying the SMMH-CreER^T2^ transgene but not the loxP-flanked *Nemo* allele served as controls (Fig. S1). To accelerate the development of atherosclerotic plaques and in order to be consistent with our earlier studies of the role of inflammatory signalling and in particular NEMO in atherosclerosis^6,8,22^, mice were subsequently placed on high fat diet (HFD) for a period of 10 weeks. At the end of this period, the development of atherosclerosis was evaluated by assessment of plaque formation in the aorta. NEMO^SMCiKO^*/ApoE*^−/−^ and *ApoE*^−/−^ mice showed similar body weight and serum levels of cholesterol and triglycerides after HFD treatment (Fig. 1a–c), indicating that SMC-specific deletion of NEMO did not affect HFD-induced obesity or basic lipid metabolism.

Histological analysis of heart sections at the level of the aortic sinus revealed significantly decreased atherosclerotic lesion development in NEMO^SMCiKO^ /*ApoE*^−/−^ mice compared to their *ApoE*^−/−^ littermates (Fig. 1d,e, pooled data from two independent experiments; the results of the individual groups are presented in Fig. S2a,b**)**. In agreement with the reduced lesion areas we observed reduced lipid deposition in the aortic arches of NEMO^SMCiKO^*/ApoE*^−/−^ mice (Fig. 1f,g). Taken together, these results demonstrated that ablation of NEMO in SMCs considerably inhibited the development of atherosclerotic plaques in *ApoE*^−/−^ mice.

### SMC-specific NEMO ablation reduced HFD-induced macrophage infiltration, collagen deposition and inflammation in aortas of ***ApoE***^−***/***−^ mice

**Figure 2:**
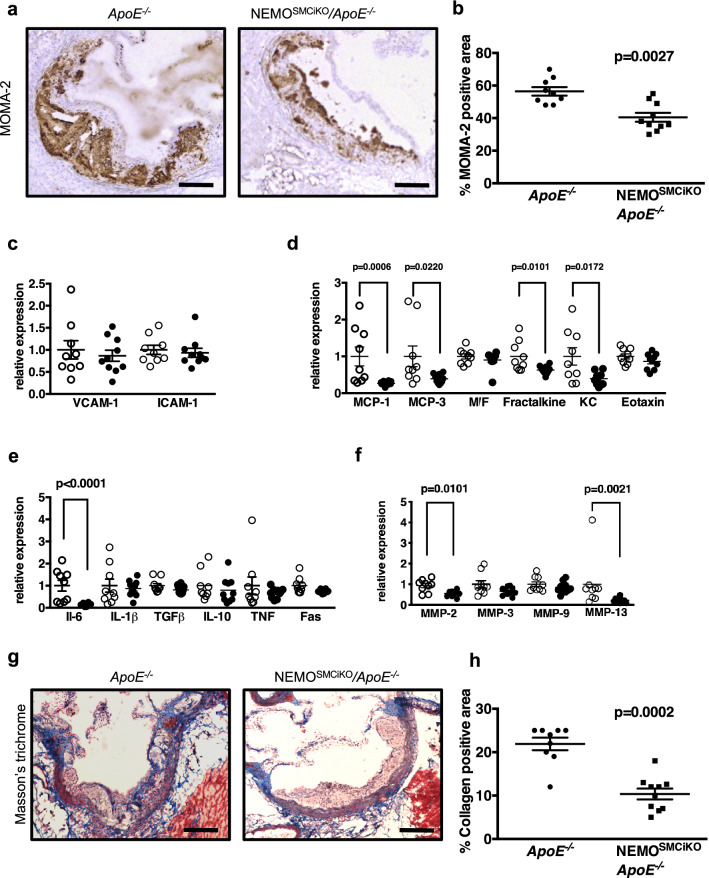
Reduced monocyte recruitment and inflammation in the aortas of *ApoE*^−*/*−^ mice with inducible deletion of NEMO in smooth muscle cells. Male NEMO^SMCiKO^/*ApoE*^−*/*−^ mice and *ApoE*^−*/*−^ mice were fed a HFD for 10 weeks before sacrifice. (**a**) Representative pictures from immunostainings of atherosclerotic lesions of *ApoE*^−*/*−^ or NEMO^SMCiKO^/*ApoE*^−*/*−^ mice with antibodies against MOMA-2. Scale bars = 0.1 mm. (**b**) Graph showing quantification of macrophage content in the lesions of *ApoE*^−*/*−^ (n = 9) or NEMO^SMCiKO^/*ApoE*^−*/*−^ (n = 10) mice (Mann–Whitney test). RNA was isolated from the aortic arches of *ApoE*^−*/*−^ (open circles, n = 9) or NEMO^SMCiKO^/*ApoE*^−*/*−^ (filled circles, n = 10) mice and the relative expression of adhesion molecules (**c**), cytokines (**d**), chemokines (**e**) and metalloproteases (**f**) was analyzed. Results represent mean ± SEM (Mann–Whitney U-test). (**g**) Representative pictures of Masson Trichrome staining in atherosclerotic lesions of *ApoE*^−*/*−^ or NEMO^SMCiKO^/*ApoE*^−*/*−^ mice. Scale bars = 0.1 mm. (**h**) Graph showing quantification of collagen content in the lesions of *ApoE*^−*/*−^ (n = 9) or NEMO^SMCiKO^/*ApoE*^−*/*−^ (n = 10) mice (Mann–Whitney test).

To address the mechanisms by which SMC-specific NEMO ablation inhibited the development of atherosclerosis, we first examined whether NEMO deficiency in SMCs affected the presence of macrophages in the plaques. Immunostaining of aortic root sections with antibodies against MOMA-2 revealed that atherosclerotic lesions of NEMO^SMCiKO^*/ApoE*^−/−^ contained considerably less macrophages compared to the lesions of their littermate *ApoE*^−/−^ mice after 10 weeks of HFD feeding (Fig. 2a,b). Therefore, SMC-specific NEMO deficiency reduced macrophage content in the plaques, suggesting that NF-κB signalling in SMCs regulates the recruitment of monocytes into developing lesions.

To dissect the mechanisms by which SMC-specific NEMO deficiency reduced macrophage accumulation in the lesions of *ApoE*^−/−^ mice and thus atherosclerosis severity we analysed the expression of adhesion molecules, chemokines, cytokines and metalloproteases in aortic arches isolated from NEMO^SMCiKO^*/ApoE*^−/−^ mice or their littermate *ApoE*^−/−^ controls after 10 weeks of HFD feeding. While the expression levels of adhesion molecules VCAM-1 and ICAM-1 did not differ between genotypes (Fig. 2c), several chemokines including MCP-1, MCP-3, fractalkine and KC were expressed at reduced levels in the aortas of NEMO^SMCiKO^*/ApoE*^−/−^ mice compared to their *ApoE*^−*/*−^ littermates (Fig. 2d). Moreover, IL-6 mRNA levels were considerably lower in the aortas of NEMO^SMCiKO^*/ApoE*^−/−^ mice, indicating reduced inflammation in the aortas of these mice (Fig. 2e). Interestingly, MMP-2 and MMP-13 that are produced by macrophages and SMCs within the atherosclerotic lesions were also expressed at significantly reduced levels in the aortas of NEMO^SMCiKO^*/ApoE*^−/−^ mice when compared to their littermate *ApoE*^−/−^ mice (Fig. 2f). Together, these results showed that ablation of NEMO in SMCs ameliorated atherosclerosis by inhibiting the expression of proinflammatory cytokines and chemokines, and reducing the accumulation of macrophages in atherosclerotic lesions.

Activation of SMCs is coupled to switching to a synthetic phenotype, which correlates with cell migration and synthesis of extracellular matrix. To test if NEMO ablation affected SMC activation and the deposition of collagen within the atherosclerotic lesions of HFD-fed mice, we performed Masson’s trichrome staining in aortic root sections of NEMO^SMCiKO^*/ApoE*^−/−^ mice and littermate *ApoE*^−/−^ mice. We found that atherosclerotic plaques of NEMO^SMCiKO^*/ApoE*^−/−^ mice contained less collagen compared to the lesions of their *ApoE*^−/−^ controls (Fig. 2g,h), arguing that NEMO ablation inhibits the phenotype switching of SMCs and the production of collagen within the plaques.

### SMC-specific NEMO ablation reduced apoptosis and necrotic core formation in atherosclerotic plaques

**Figure 3:**
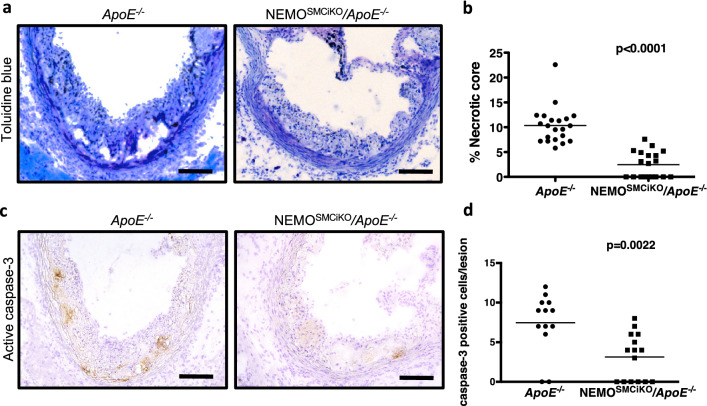
Reduced necrotic core and apoptosis in the aortas of *ApoE*^−*/*−^ mice with inducible deletion of NEMO in smooth muscle cells. Male *ApoE*^−*/*−^ and NEMO^SMCiKO^/*ApoE*^−*/*−^ mice were fed a HFD for 10 weeks before sacrifice. (**a**) Representative pictures from atherosclerotic lesions of *ApoE*^−*/*−^ or NEMO^SMCiKO^/*ApoE*^−*/*−^ mice, where the necrotic acellular areas can be seen. Scale bars = 0.1 mm. (**b**) Graph showing quantification of the percentage of the necrotic area within the lesions of *ApoE*^−*/*−^ or NEMO^SMCiKO^/*ApoE*^−*/*−^ mice (Mann–Whitney test). (**c**) Representative pictures from atherosclerotic lesions of *ApoE*^−*/*−^ or NEMO^SMCiKO^/*ApoE*^−*/*−^ mice, stained with antibodies against active caspase-3. Scale bars = 0.1 mm. (**d**) Graph showing quantification of the percentage of apoptotic cells within the lesions of *ApoE*^−*/*−^ or NEMO^SMCiKO^/*ApoE*^−*/*−^ mice after 10 weeks on HFD (Mann–Whitney test).

Our results indicated that deletion of NEMO in SMCs inhibited atherosclerosis development mainly by reducing macrophage accumulation in atherosclerotic plaques. During atherosclerosis development death of macrophages and reduced clearance of apoptotic debris correlates with accelerated disease development^23^. We therefore evaluated apoptosis and the presence of necrotic core in atherosclerotic lesions of NEMO^SMCiKO^*/ApoE*^−/−^ or *ApoE*^−/−^ mice. Atherosclerotic plaques in *ApoE*^−/−^ mice contained larger necrotic areas compared to the plaques found in NEMO^SMCiKO^*/ApoE*^−/−^ mice (Fig. 3a,b, pooled data from two independent experiments; the results of the independent groups are presented in Fig. S2c,d**)**. In line with the reduced necrotic core formation, we observed reduced number of cells staining positive for cleaved/activated caspase-3 in the lesions of NEMO^SMCiKO^*/ApoE*^−/−^ mice compared to their littermate *ApoE*^−/−^ controls (Fig. 3c,d). Therefore, SMC-specific NEMO ablation reduced apoptosis and necrotic core formation in atherosclerotic plaques.

### NEMO deficiency inhibits smooth muscle cell activation in response to oxidised LDL and inflammatory stimulus

**Figure 4:**
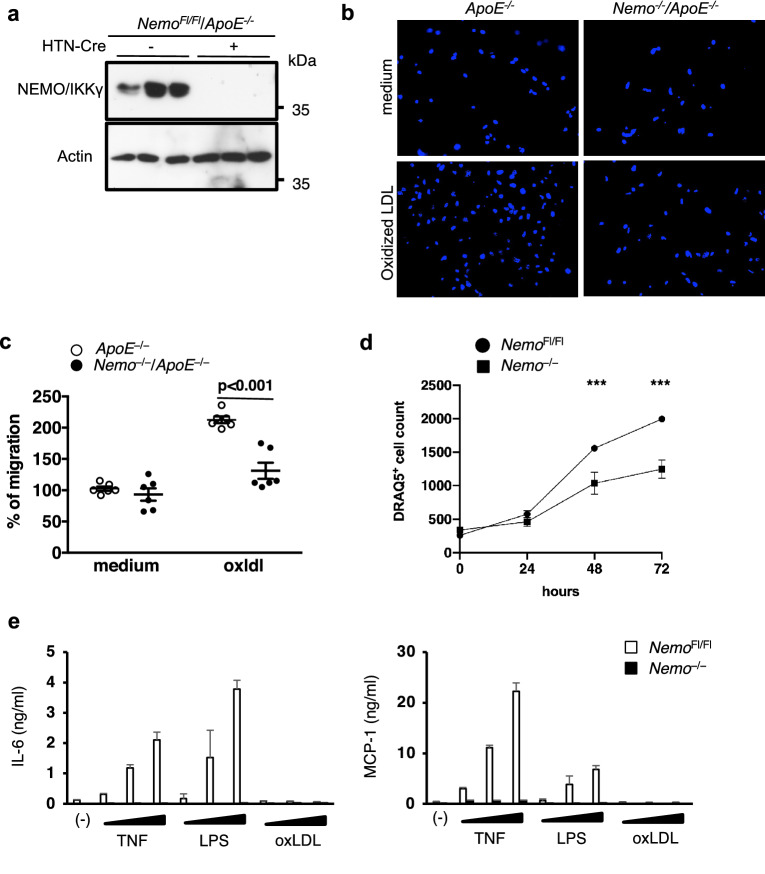
Deletion of NEMO in smooth muscle cells inhibits their activation upon oxidized LDL stimulation and inflammatory stimulus. (**a**) Presentation of the deletion efficiency in vitro using HTN-Cre mediated excision of the NEMO allele. Three independent isolations of smooth muscle cells are depicted. Uncropped blots are presented in Supplementary Fig. 4. (**b**) Representative pictures of migrating smooth muscle cells (Transwell assay) after staining with DAPI. (**c**) Graph showing quantification of the migration of *ApoE*^−*/*−^* or Nemo*^−*/*−^*/ApoE*^−*/*−^ smooth muscle cells towards oxidized LDL. The graph represents the combined results of two independent experiments performed with *ApoE*^−*/*−^* or Nemo*^−*/*−^*/ApoE*^−*/*−^ smooth muscle cells from 3 independent isolations (2-way ANOVA with Bonferroni post-hoc test) (**d**) Graph showing quantification of the proliferation of *Nemo*^*Fl/Fl*^ or *Nemo*^−*/*−^ smooth muscle cells. Data are presented as mean ± SEM and are representative of three independent experiment with similar results. (*t* test, ***p < 0.05). (**e**) *Nemo*^*Fl/Fl*^ or *Nemo*^−*/*−^ smooth muscle cells were stimulated with TNF (0.1, 1, 10 ng/ml), LPS (0.1, 1, 10 ng/ml), and oxidized LDL (10, 30 100 µg/ml) or left unstimulated. At 24 h after stimulation, the supernatants were collected, and the concentration of IL-6 and MCP-1 were determined by ELISA. Data are presented as mean ± SD and are representative of three independent experiments with similar results.

Modified lipids were previously shown to bind TLRs^24^ and activate SMCs^25^. We have previously demonstrated that modified lipids can activate NF-κB via TLR4-TRAF6-dependent signalling in endothelial cells and macrophages^6^. We therefore reasoned that NEMO deficiency reduced atherosclerotic plaque formation by inhibiting modified lipid-induced activation of SMCs. To address this hypothesis we isolated SMCs from aortas of NEMO^Fl/Fl^/*ApoE*^−*/*−^ mice and induced deletion of NEMO in vitro by application of HTN-Cre (Fig. 4a). To investigate if the absence of NEMO could affect the activation of SMCs by modified lipids we examined oxidized LDL-induced responses^26,27^ in *ApoE*^−*/*−^ and *NEMO*^−*/*−^*/ApoE*^−*/*−^ SMCs. To test if NEMO deficiency affected the modified lipid-induced migration/chemotaxis of *ApoE*^−*/*−^ SMCs we compared the ability of *NEMO*^−*/*−^*/ApoE*^−*/*−^ and *ApoE*^−*/*−^ SMCs to migrate towards oxidized LDL using the Transwell assay. This experiment showed that *NEMO*^−*/*−^*/ApoE*^−*/*−^ SMCs exhibited impaired migration towards oxidized LDL compared to *ApoE*^−*/*−^ SMCs (Fig. 4b,c**)**, demonstrating that NEMO-dependent canonical NF-κB signalling is required for oxidized LDL-mediated SMC migration. As migration of SMCs is a key event for the development of atherosclerotic lesions, the finding that NEMO deficiency inhibited oxidized LDL-induced SMC migration are in line with the atheroprotective effect of SMC-specific NEMO deficiency in vivo.

During the development of atherosclerotic lesions SMCs also demonstrate increased proliferative capacity, which is coupled to their migration. To assess if NEMO deficiency affected SMC proliferation, we incubated *Nemo*^−*/*−^ SMCs and *Nemo*^*Fl/Fl*^ SMCs for 72 h and evaluated their proliferative capability. We observed that *Nemo*^−*/*−^ SMCs proliferated less compared to *Nemo*^*Fl/Fl*^ SMCs (Fig. 4d). In addition, and in line with our results in vivo, production of IL-6 and MCP-1 were reduced in *Nemo*^−*/*−^ SMCs compared to *Nemo*^*Fl/Fl*^ SMCs upon stimulation with inflammatory mediators, such as TNF and LPS (Fig. 4e). Whereas reduced viability in response to stimulation with TNF could partly contribute to the diminished cytokine production in *Nemo*^−*/*−^ SMCs, these cells did not undergo cell death after LPS stimulation (Supplementary Fig. 3), suggesting that NEMO deficiency prevents NF-κB-dependent cytokine production in SMCs. These results are in agreement with our in vivo observation that ablation of NEMO in SMCs inhibits inflammation in aorta and atherosclerosis development.

### NEMO deficiency inhibits smooth muscle cell phenotype switching

**Figure 5:**
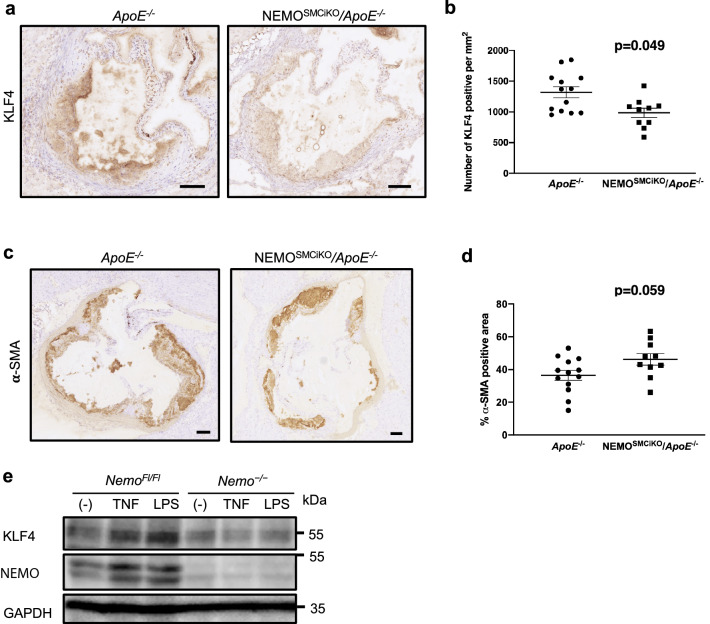
Deletion of NEMO in smooth muscle cells inhibits their phenotype switching. Representative pictures from immunostainings of atherosclerotic lesions of *ApoE*^−*/*−^ or NEMO^SMCiKO^/*ApoE*^−*/*−^ mice with antibodies against KLF4 (**a**) and α-SMA (**c**). Scale bars = 0.1 mm. Graph showing quantification of KLF4 positive cells (**b**) and α-SMA content (**d**) in the lesions of *ApoE*^−*/*−^ (n = 13) or NEMO^SMCiKO^/*ApoE*^−*/*−^ (n = 10) mice (Mann–Whitney test). (**e**) Cultured smooth muscle cells were stimulated with TNF (1 ng/ml) and LPS (10 ng/ml) or left unstimulated in starved medium for 48 h. Total protein lysates of smooth muscle cells were subjected to western blot analysis of KLF4 and NEMO protein expression. Glyceraldehyde 3-phosphate dehydrogenase (GAPDH) was used as a loading control. Uncropped blots are presented in Supplementary Fig. 4.

In response to inflammatory stimulus SMCs can undergo phenotype switching from contractile cells to synthetic cells possessing a pro-inflammatory matrix remodelling phenotype^16^. Krüppel-like factor 4 (KLF4) was shown to regulate transition of SMCs towards a macrophage-like pro-inflammatory phenotype, whereas SMC-specific KLF4 deficiency reduced atherosclerotic plaque development in *ApoE*^−*/*−^ mice^28^. We therefore evaluated the expression of KLF4 as well as α-SMA, a marker of smooth muscle cells, in atherosclerotic plaques of NEMO^SMCiKO^*/ApoE*^−/−^ mice and littermate controls. We found that the expression of KLF4 was significantly downregulated whereas the expression of α-SMA was mildly upregulated in plaques of NEMO^SMCiKO^*/ApoE*^−/−^ compared to *ApoE*^−/−^ mice (Fig. 5a–d). Together with the results showing reduced macrophage staining in plaques from NEMO^SMCiKO^*/ApoE*^−/−^ (Fig. 2a,b), these findings suggested that NEMO deficiency may negatively regulate phenotype switching of SMCs towards a synthetic phenotype. In line with this, *Nemo*^−*/*−^ SMCs showed considerably lower KLF4 expression upon stimulation of TNF and LPS (Fig. 5e). Taken together, the results from our in vivo and in vitro experiments indicate that NEMO-dependent signalling is required for the activation and phenotype switching of SMCs in response to inflammatory stimuli, suggesting that canonical NF-κB signalling controls critical biological functions of SMCs that are relevant for atherosclerotic plaque formation and progression.

## Discussion

The development and growth of an atherosclerotic plaque is the result of a chronic non-resolving inflammatory response involving the activation of resident cells of the vascular wall, primarily endothelial cells and smooth muscle cells, and a constant influx and entrapment of monocytes/macrophages within the plaque. Although the contribution of macrophages and endothelial cells in atherosclerosis is well documented, the in vivo role of SMCs in regulating the onset and progression of atherosclerotic plaque development has remained less well understood. In the present study, we used SMC-specific targeting of NEMO to address the function of canonical NF-κB signalling in the pathogenesis of atherosclerosis in vivo. Our results revealed that inducible deletion of NEMO in SMCs significantly inhibited HFD-induced atherosclerosis in *ApoE*^−*/*−^ mice. NEMO deficiency in SMCs resulted in reduced plaque size, decreased number of macrophages and reduced apoptosis and necrotic core formation in atherosclerotic lesions. In addition, SMC-specific NEMO deficiency also inhibited the induction of a number of inflammatory mediators in aortas of HFD fed *ApoE*^−*/*−^ mice and in cultured SMCs, suggesting that SMCs constitute an important source of NF-κB-dependent cytokines and chemokines that regulate inflammation in the vascular wall. Collectively, these results provided in vivo experimental evidence that NEMO-dependent NF-κB activation in SMCs is critically involved in the pathogenesis of atherosclerosis.

Our results are in agreement with a previous report showing that IKK2-mediated NF-κB activation in SMCs regulates atherosclerosis development and obesity^29^. In this study however, the SM22a-Cre transgene employed deleted IKK2 not only in SMCs but also in adipocytes. IKK2 deficiency in adipocytes affected basic metabolic functions rendering these mice resistant to HFD-induced obesity and obesity-associated metabolic disorders, which could indirectly affect the development of atherosclerosis, rendering the interpretation of these experiments more complicated. In our study, inducible deletion of NEMO was achieved using the SMMH-CreER^T2^ transgene expressing Cre recombinase specifically in SMCs of the large organized vessels^21^. Indeed, tamoxifen administration resulted in NEMO deletion specifically in SMCs but not in white adipose tissue or liver of the SMMH-CreER^T2^/*Nemo*^Fl/Fl^ mice (Fig. S1b,c). Therefore, our experiments complement the study by Sui et al.^29^ by demonstrating that SMC-specific NEMO ablation inhibits atherosclerosis in the absence of a concomitant metabolic defect.

Our in vitro mechanistic studies indicate that NEMO is a critical regulator of SMC proliferation and migration, which are important characteristics of the phenotypic switching triggered by KLF4 during the development of atherosclerosis^16,28^. In agreement with this, we showed that KLF4 expression in SMCs was upregulated in a NEMO dependent manner upon inflammatory stimulation. Our results are also consistent with a study demonstrating that inhibition of NF-κB by the NBD peptide reduced proliferation and migration of SMCs in vitro^30^. In regions of the vasculature that are prone to develop atherosclerotic lesions NF-κB activation is most likely induced as a result of a variety of upstream signals like TLRs^31,32^, TNF^33,34^, IL-1β^35,36^, or angiotensin II^37,38^ that mediate cell proliferation and migration but may also induce inflammation or apoptosis of SMCs^39^. Indeed, our results showed that NEMO-deficient SMCs produced reduced amounts of inflammatory cytokines and chemokines compared to *Nemo*^Fl/Fl^ SMCs upon stimulation with LPS or TNF. Oxidized LDL has been reported to activate TLR4-dependent inflammatory cytokine expression in SMCs^40^, which could act in an autocrine manner to induce SMC activation. Since activation of SMCs is an early event in the onset of atherosclerosis, detected already before or at the same time when monocyte/macrophage infiltration is observed, it is possible that sensing of modified lipids within the intima provides the signal for the initial activation of SMC in early lesions. Production of inflammatory mediators by SMCs themselves, but also infiltrating monocytes and endothelial cells, could further induce activation and phenotype switching of SMCs, contributing to the acceleration of atherosclerosis progression.

The role of the NF-κB pathway in the regulation of immune and inflammatory responses is well established and numerous studies have suggested the potential importance of NF-κB as a therapeutic target for vascular diseases. Several studies indicate that the inhibition NF-κB activation, mediated by upstream receptors like TLRs, TNFR1, or IL-1R might be of a potential therapeutic interest for the treatment of atherosclerosis^41,42^. Nevertheless, our earlier experiments revealed that TLR/NF-κB signalling exhibits cell-specific functions in atherosclerosis. Inhibition of TLR/NF-κB signalling specifically in vascular endothelial cells protected *ApoE*^−/−^ mice from atherosclerosis by preventing the expression of proinflammatory factors and the recruitment of monocytes to the developing plaques^6,8^. In contrast, we showed that TRAF6-dependent and IKK2-dependent signalling in macrophages is atheroprotective in *ApoE*^−*/*−^ mice, although the role of the TLR/NF-κB signalling in this cell type might be more complex^5,6^. Our results presented here showed that inhibition of NEMO-dependent NF-κB signalling in SMCs protected mice from the development of atherosclerosis, similar to our findings in endothelial cells. Together, these studies suggest that targeting IKK-mediated NF-κB activation in resident stromal cells of the vascular wall (endothelial cells and SMCs) could prove beneficial for the treatment of atherosclerosis.

## Methods

### Mice and diet

Mice with conditional loxP-flanked NEMO alleles have been previously described^20^ and were crossed with SMMH-CreER^T2^ transgenic mice^21^ to generate mice with tamoxifen-inducible SMC-specific NEMO deficiency. In order to allow studying the effect of NEMO deficiency on atherosclerosis, these mice were then crossed with *ApoE*^−*/*−^ mice, which constitute a widely accepted mouse model of the disease^43^. All mice were in the C57Bl/6 background. For induction of Cre activity, male mice carrying the SMMH-CreER^T2^ transgene were fed a tamoxifen-containing diet for 6 weeks starting at the age of 6 weeks. Subsequently, the mice were placed on a western–type diet (Harland Tekland, TD88137 high fat diet) for 10 weeks, to accelerate the development of atherosclerotic lesions, as previously described^6,8,22^. All in vivo experiments were performed twice, by analyzing two independent groups of littermate mice. At the end of the experiment the animals were humanely sacrificed according to approved animal protocols and tissues and sera were collected for further analysis. All animal procedures were conducted in accordance with European, national, and institutional guidelines and were approved by the responsible local governmental authorities (Landesamt für Natur, Umwelt und Verbraucherschutz Nordrhein-Westfalen) and are reported in accordance with ARRIVE guidelines.

### Lipid analysis

Serum cholesterol levels were measured after overnight fasting using the PAD-CHOL reagent (Roche) according to the manufacturer’s instructions. Triglyceride levels in the serum were determined by a commercial available kit (AbCam).

### Immunostainings

Frozen sections of the aortic root were fixed in ice-cold acetone for 10 min, dried under a ventilator, and washed with PBS. Sections were blocked in 4% FCS with Avidin D solution (Avidin/Biotin Blocking Kit; Vector Laboratories) for 30 min. Primary antibodies were anti-mouse macrophages/monocytes (MCA519GT, Serotec), anti- active caspase 3 (AF835, R&D Systems), anti- α-SMA (A2547, Sigma-Aldrich), and anti- KLF4 (NBP2-24749, Novus Biological). Biotinylated secondary antibodies, ABC Kit Vectastain Elite, and DAB substrate (PerkinElmer, Vector, and Dako) were used. After counterstaining with haematoxylin sections were mounted with Entellon (MERCK) mounting medium. Stained area was measured using Adobe Photoshop or QuPath^44^.

### Histology of plaques and lesion size

Consecutive 7 μm sections of the heart in the atrioventricular valve region were collected and stained with toluidine blue, as described previously^5^. For morphometric analysis lesion size was measured on four consecutive sections in 42 μm intervals using Adobe Photoshop.

### En face staining

Sudan IV staining and *en face* analysis of atherosclerotic lesions were performed as described previously^45^. Areas that were stained for lipids were quantified using Adobe Photoshop.

### Oxidation of human LDL

CuSO_4_ oxidation of human LDL (AppliChem) was performed at 37 °C according to standard protocols and as previously described^5^.

### Isolation and culture of SMCs from murine aortas

Mice were sacrificed and the heart was perfused through the apex with sterile HBSS. All organs, except the heart, were removed to allow a clear view of the aorta. The fat tissue around the aortic region was removed, aortas were isolated and placed in ice cold HBSS, washed twice and placed in enzyme solution (1 mg/ml collagenase, 0.3 mg/ml trypsin inhibitor, 0.75U/ml elastase, 20% FCS in DMEM). After incubation (10 min, 37 °C), aortas were washed in DMEM/F12 medium. The adventitia was stripped off and the aorta was opened longitudinally with scissors. The aorta was further washed in DMEM/F12 medium to removed blood clots. The endothelial cell layer was removed by gently scrapping the inside of the vessel with forceps and the aortas were further washed in equilibrated DMEM/F12 medium. Final digestion was performed in enzyme solution (45 min, 37 °C, 5% CO_2_). Cells were collected by centrifugation, washed 3 times with DMEM/F12 medium and plated in one gelatin-coated 24-well. Cells were passaged when 95% confluent and used in passages 7–12. Deletion of NEMO in SMCs was performed at passage P5 by incubation of cells with HTN-Cre^46^ in serum free medium for 16–20 h as previously described^6,22^.

### Migration assays

Chemotaxis of SMCs toward oxidized LDL was performed in a 24-well microchemotaxis chamber (Costar, #3422), using polycarbonate membranes with 8 μm pores similarly to previously described^47^. SMCs were harvested and plated at a concentration of 10^4^ cells/0.1 ml of DMEM containing 2% FCS in the upper chamber of the Transwell. The bottom chamber was filled with 0.6 ml of DMEM containing 2% FCS. Wherever appropriate oxidized LDL was supplemented to the bottom chamber at the concentration of 100 μg/ml. The upper chamber was loaded with 10^4^ cells and incubated for 8 h at 37 °C. After completion of the incubation, the filters were fixed with 4% saline-buffered formalin, non-migrating cells (upper side of the filters) were scraped off the filter and nuclei of the migrating cells (lower side of the filters) were stained with DAPI. The cells that migrated through the filter were counted at 5 different areas of each filter at 20× magnification. Results were normalized to the non-treated *ApoE*^−*/*−^ cells and represent two independent experiments where cells from three different isolations of SMCs were used.

### Proliferation assays

SMCs were plated in 96-well plate at the concentration of 10^4^ cells/200 μl medium/well for 16 h before assays. The number of cells was assessed at 24, 48 and 72 h. Cells were stained with DRAQ5 (65-0880-96, Thermo Fisher Scientific/Life Technologies) according to the manufacturer’s instruction and quantified using the IncuCyte bioimaging platform (Essen); two to four images per well were captured, analysed and averaged.

### Cell death assays

SMCs were seeded in 96-well plates (10^4^ cells per well) 16 h before treatment. On the day of the experiment, indicated amounts of recombinant mouse TNF (VIB Protein Service Facility) or LPS (ALX-581-010-L002, Enzo) were added to cells. Cell death assays were performed using the IncuCyte bioimaging platform. Cell death was measured by the incorporation of DiYO-1 (ABD-17580, AAT Bioquest).

### In vitro phenotype switch assays

SMCs were plated in 12-well plate at the concentration of 10^5^ cells/2 ml medium/well for 16 h before stimulation. Cells were stimulated with TNF (1 ng/ml) or LPS (10 ng/ml) for 48 h in starved medium and then collected for western blot.

### Immunoblotting

Cell lysates were denatured in 2× Laemmli buffer. The proteins samples were subsequently boiled at 95 °C for 10 min and separated by SDS–PAGE. Separated proteins were transferred to PVDF membranes. Incubation of the membranes with primary antibodies was performed in TBS supplemented with 0.1% Tween-20 (v/v) and 2.5% (w/v) BSA. The immunoblots were incubated overnight with primary antibodies against KLF4 (NBP2-24749, Novus Biological, 1:200), NEMO (homemade rabbit polyclonal serum), and GAPDH (NB300-221, 1:5000).

### Quantitative real-time PCR

RNA was isolated from aortas and SMCs using Trizol-reagent (Invitrogen) and RNeasy columns (QIAGEN). RNA (1 μg) was used for reverse transcription with SuperScript III reverse transcriptase (Invitrogen). The reaction was topped up to 200 μl with water, and 2 μl were used for quantitative real-time PCR reaction with TaqMan qPCR Kit (Thermo Fischer Scientific) from Eurogentec. Standardization was performed with primers Tata-box protein. VCAM-1; Mm00449197_m1, ICAM-1; Mm00516023_m1, MCP-1; Mm00441242_m1, MCP-3; Mm00443113_m1, MIF; Mm01611157_gH, Fractalkine; Mm00436454_m1, KC; Mm00433859_m1, Eotaxin; Mm00441238_m1, IL-6; Mm00446190_m1, IL-1β; Mm00434228_m1, TGFβ; Mm03024053_m1, IL-10; Mm00439616_m1, TNF; Mm00443258_m1, Fas; Mm00433237_m1, MMP-2; Mm00439498_m1, MMP-3; Mm00440295_m1, MMP-9; Mm00442991_m1, MMP-13; Mm00439491_m1, Tata-box protein; Mm01277042_m1.

### Cytokine, chemokine assays

The concentration of IL-6 and MCP-1 in the supernatant from SMC cultures were measured using mouse IL-6 and MCP-1 ELISA kit (Thermo Fischer Scientific).

### Statistical analysis

Statistical analyses were performed using Prism (GraphPad Software Inc., San Diego, CA). Data are expressed as mean ± SEM unless otherwise specified. Statistical significance was assessed using the Mann–Whitney test for 2-group comparisons. For multiple comparisons 2-way ANOVA with repeated measures followed by *t* test with Bonferroni correction was used. Differences were considered statistically significant at a value of *p* < 0.05.

## Supplementary Information

Supplementary Figures.

## Data Availability

The datasets used and/or analyzed during the current study are available from the corresponding author on reasonable request.
