# Supplementary material for: Smooth muscle cell specific NEMO deficiency inhibits atherosclerosis in ApoE−/− mice
Source: Sci Rep. 2022 Jul 22;12:12538. doi: 10.1038/s41598-022-16737-8 (PMC9307802; doi:10.1038/s41598-022-16737-8)
Supplement: Supplementary file 1 — Supplementary Figures. [file 41598_2022_16737_MOESM1_ESM.pdf]

Smooth muscle cell specific NEMO deficiency inhibits atherosclerosis in ApoE$^{-/-}$ mice

Takashi Imai [1,2,3], Trieu-My Van [1,2,3], Manolis Pasparakis [1,2,3], Apostolos Polykratis[1,2,3]

[1]Institute for Genetics, [2]Centre for Molecular Medicine Cologne, (CMMC) and [3]Cologne Excellence Cluster on Cellular Stress Responses in Aging-Associated Diseases (CECAD), University of Cologne, 50931, Cologne, Germany

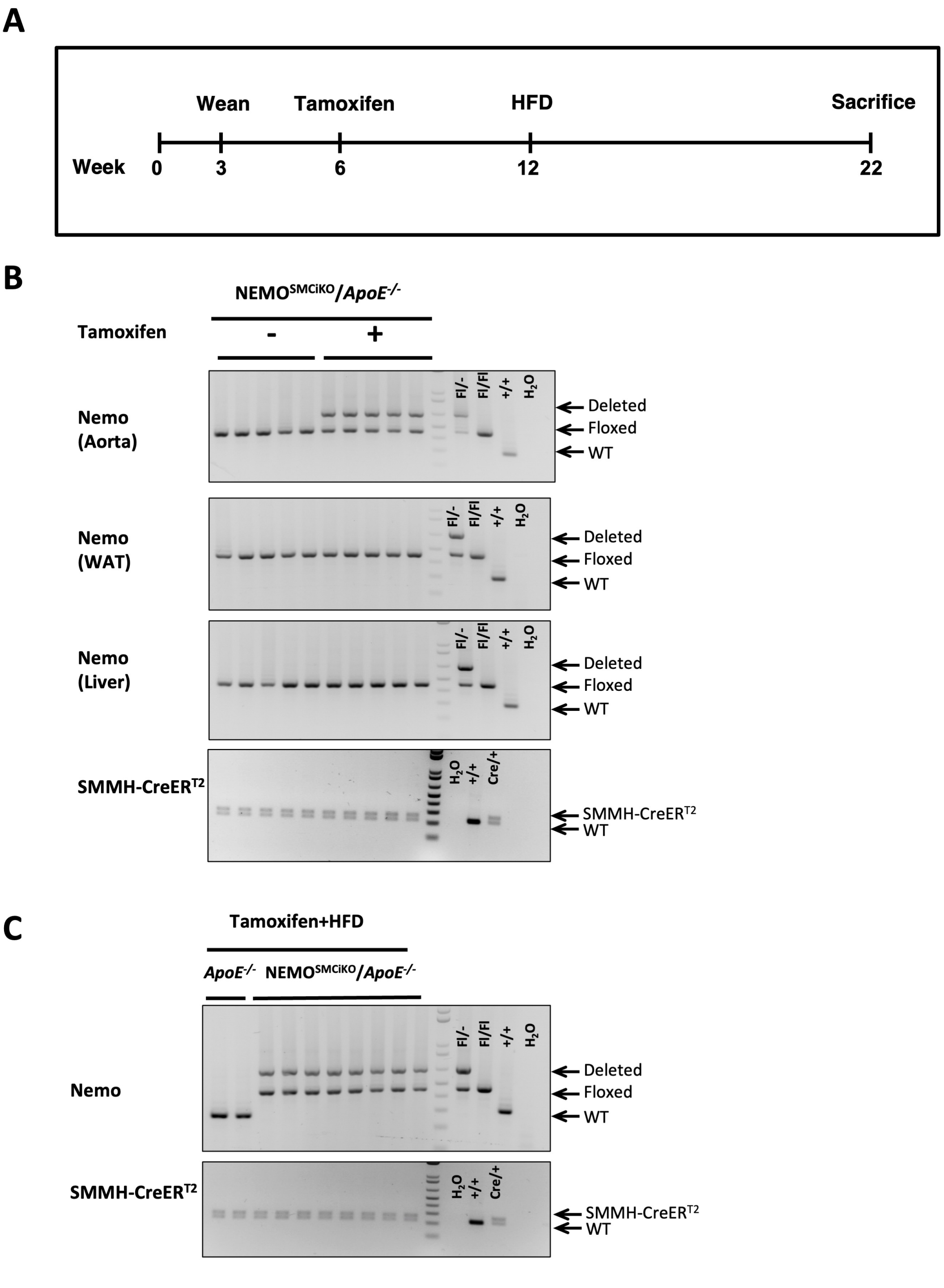

Supplementary Figure 1. Inducible deletion of NEMO specifically in smooth muscle cells. (a) Graph depicting the experimental approach followed to investigate the role of NEMO in smooth muscle cells during the development of atherosclerosis. (b) Detection of NEMO alleles by PCR in the aortas, white adipose tissue (WAT), and liver of NEMO^{SMCiKO}/*ApoE*$^{-/-}$ mice that were fed a normal diet or tamoxifen-containing diet for 6-weeks starting at the age of 6 weeks. The PCRs were performed in samples from the respective tissues 2 weeks after the end of the tamoxifen treatment. (c) Detection of NEMO alleles by PCR in the aortas of *ApoE*$^{-/-}$ or NEMO^{SMCiKO}/*ApoE*$^{-/-}$ mice that were treated with a tamoxifen-containing diet for 6-weeks starting at the age of 6 weeks and with a HFD for an additional period of 10 weeks. The PCRs were performed in samples from aortas isolated at the day of the sacrifice.

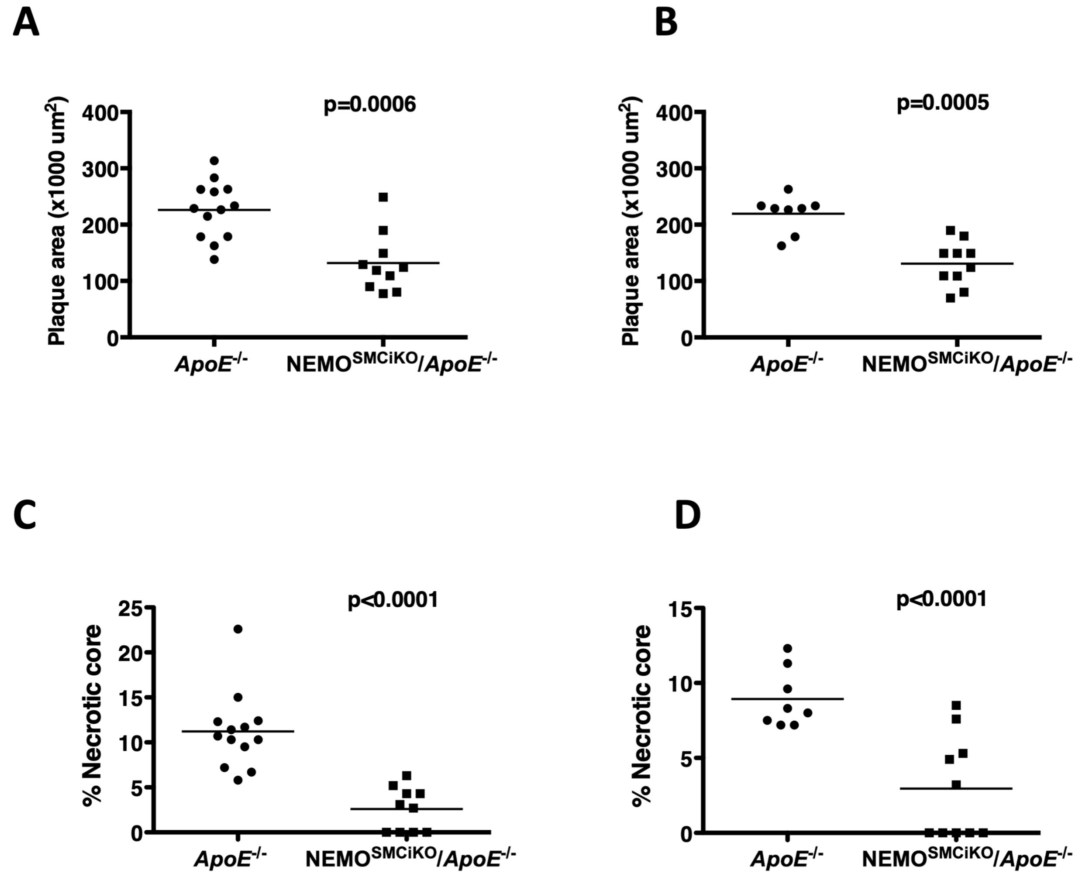

Supplementary Figure 2. Deletion of NEMO in smooth muscle cells inhibits atherosclerosis. Graphs showing the results of two independent experiments on the lesion area (a-b) or the percentage of necrotic core in the atherosclerotic plaques (c-d) of *ApoE*$^{-/-}$ or NEMO^{SMCiKO}/*ApoE*$^{-/-}$ mice after 10 weeks on HFD (Mann-Whitney test).

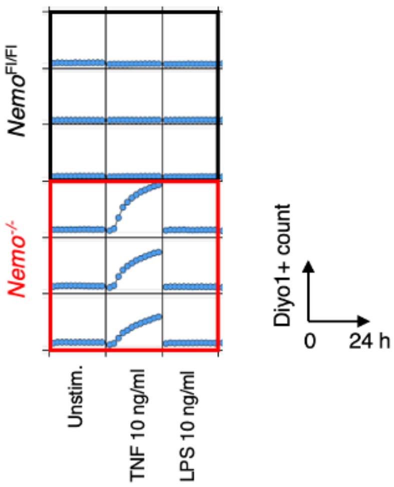

Supplementary Figure 3. TNF, but not LPS, induced cell death in *Nemo*$^{-/-}$ SMCs.
IncuCyte graphs showing one representative of three independent experiments measuring cell death in *Nemo*$^{Fl/Fl}$ or *Nemo*$^{-/-}$ SMCs in response to TNF or LPS stimulation. Dead cells are revealed by staining with Diyo1.

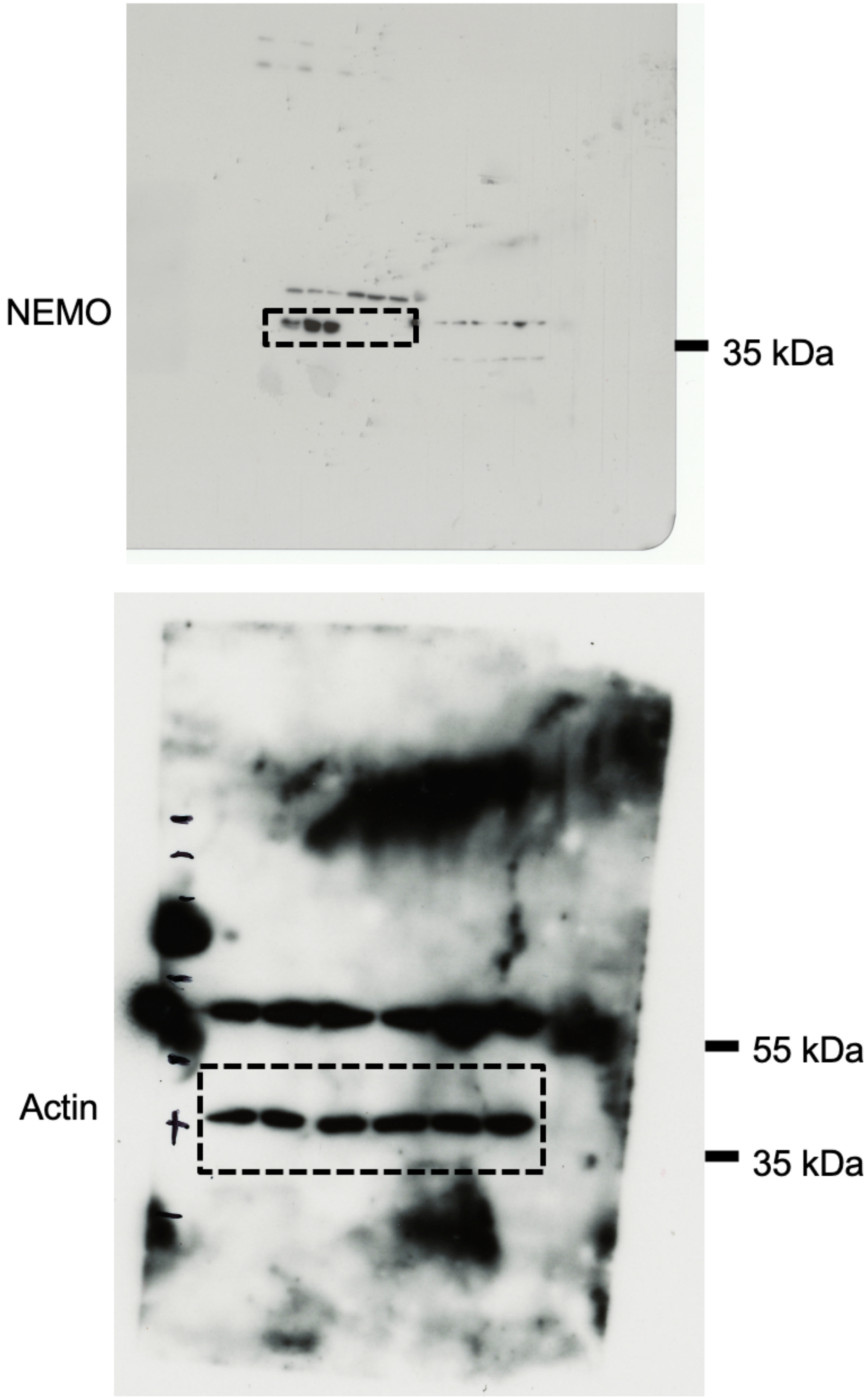

Figure 4a

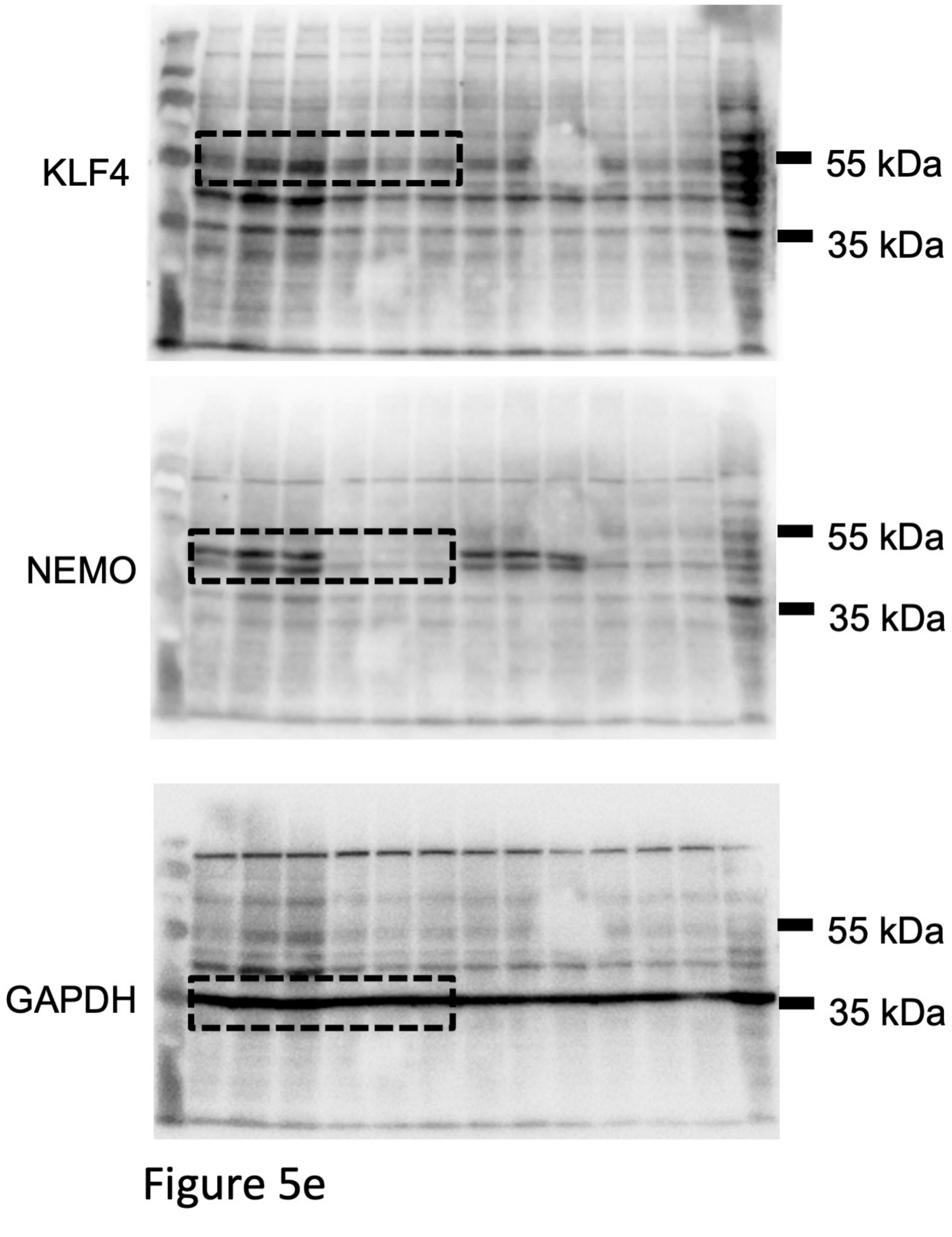

Supplementary Figure 4. Uncropped images of the immunoblots shown in Figure 4a and Figure 5e. In Figure 4a, the same extracts were run on two separate gels side by side and probed with anti-NEMO (above) or anti-Actin (bottom) antibodies. In Figure 5e, the same membrane was re-probed with antibodies against KLF4, NEMO and GAPDH.
